# A meningoencephalitis outbreak associated with echovirus type 18 (E18) in south-western Hungary in mid-2023

**DOI:** 10.1007/s00705-024-06166-5

**Published:** 2024-11-04

**Authors:** Károly Takáts, Benigna Balázs, Ákos Boros, Dávid Sipos, Zoltán Péterfi, Márk Harmat, Dávid Varga, Zita Zengő-Bedő, Péter Pankovics, Gábor Reuter

**Affiliations:** 1 Department of Medical Microbiology and Immunology, Medical School, University of Pécs, Szigeti út 12, Pécs, H-7624 Hungary; 2 Department of Internal Medicine, Medical School, University of Pécs, Pécs, Hungary; 3 Department of Neurology, Medical School, University of Pécs, Pécs, Hungary; 4 Department of Emergency Medicine, Medical School, University of Pécs, Pécs, Hungary

## Abstract

Echovirus type 18 (E18) is a member of the genus *Enterovirus* of the family *Picornaviridae*. In this study, we investigated the characteristics of E18 infections in hospitalized adults with meningoencephalitis that occurred during an unusual epidemic in south-western Hungary in mid-2023. Five (6.1%) out of 82 cerebrospinal fluid specimens that were tested were positive for an enterovirus, four of which were E18 (OR372160 and PP861087-PP861090). Headache (100%), fever (75%), retrobulbar pain (50%), nausea (50%), joint/limb pain (50%), exanthema, photophobia, and vomiting were the most common symptoms. Sequence analysis showed that these viruses were related to unpublished emerging E18 strains from France (2022/2023) and China (2019/2020). Further study is necessary to monitor the circulation of epidemic/pandemic E18 variants over time.

**Supplementary Information:**

The online version contains supplementary material available at 10.1007/s00705-024-06166-5.

Enteroviruses (family *Picornaviridae*) are small non-enveloped viruses with a 7.2- to 8.5-kb-long, positive-sense, single-stranded RNA genome. The genus *Enterovirus*, which includes coxsackieviruses, echoviruses, rhinoviruses, and polioviruses, currently consists of 15 species [1, 2].

Enteroviruses are common infectious agents that are transmitted throughout the human population, mainly by the faecal-oral or respiratory route [3]. Enteroviruses replicate first in epithelial cells of the oropharynx and/or in the mucosa of the intestines, after which they can be carried by the bloodstream to reach different target organs. Most enterovirus infections are asymptomatic, but in rare cases, they can cause severe organ-specific syndromes (e.g., hepatitis, myocarditis, meningoencephalitis, exanthema, conjunctivitis, etc.). Enteroviruses, especially members of the species *Enterovirus betacoxsackie*, are known to cause infections of the central nervous system (CNS), resulting in aseptic meningitis, encephalitis, meningoencephalitis, and/or myelitis [4].

Echovirus type 18 (E18) [5], a member of the species *Enterovirus betacoxsackie*, was discovered in the United States in 1955 in a patient with diarrhoea [6]. Since then, E18-associated cases and outbreaks have been reported throughout the world, including Australia in 1968–1969 [7], Japan in 1981–1991 [8–10], France in 2003–2008 [11–14], Taiwan in 2006 [15], Russia in 2007 [16], Germany in 2010 [17], the Netherlands in 2011 [18], Tunisia in 2011–2013 [19], Spain in 2013 [20], Northern West Bank, Palestine, in 2017 [21], South Korea in 2011–2020 [22], and China in 2014–2024 [23–30]. The available epidemiological data indicate that the majority of the symptomatic individuals were neonates and children under the age of 13. The most common clinical manifestations associated with E18 were gastroenteritis (diarrhoea), exanthema, pharyngitis, pneumonia, meningitis, encephalitis, and neonatal sepsis-like disease [7, 8, 15, 23, 31]. The majority of the reported E18 infections in Europe occurred before 2013 in France [11–14], the European part of the Russian Federation [16], Germany [17], the Netherlands [18], and Spain [20]. Interestingly, since 2013, the number of reported E18 infections in Europe has decreased significantly [32], and the limited information we have is mostly available from unpublished data. E18 has been reported only once in Hungary: during surveillance of the circulating enterovirus types between 2010 and 2018, one of the 4080 specimens tested positive for E18 in 2016 [33].

Here, we report an unusual series of E18 infections in epidemic form associated with aseptic meningoencephalitis in hospitalized adults in south-western Hungary in mid-2023.

Inpatients with a suspected clinical diagnosis of CNS infection (acute encephalitis and/or meningitis) were examined at the Clinical Centre, University of Pécs (Pécs, Hungary), between January 1 and December 31, 2023. The Clinical Centre serves Baranya County, which had a population of 355,315 in 2023 (https://www.ksh.hu/stadat_files/nep/hu/nep0034.html). CSF sampling took place approximately 3 days (between 1 and 6 days) after the onset of clinical symptoms. Cerebrospinal fluid (CSF) samples were tested for diagnostic purposes by bacterial culture and a syndrome-specific multiplex PCR-based assay (Meningitis/Encephalitis Panel, BioFire FilmArray, bioMérieux, France) according to the manufacturer’s instructions. The latter identifies the most common viral (enterovirus, CMV, HHV1/2, HHV6, VZV, and human parechovirus), bacterial (*Escherichia coli* K1, *Haemophilus influenzae*, *Listeria monocytogenes*, *Neisseria meningitidis*, *Streptococcus agalactiae*, and *Streptococcus pneumoniae*), and fungal (*Cryptococcus neoformans/gatti*) pathogens that cause CNS infections (https://www.biofiredx.com/products/the-filmarray-panels/filmarrayme). Each molecular test was preceded by a patient-centred telephone consultation between the clinician and the medical microbiologist and was only carried out in clinically substantiated and controlled cases. The health data collection authorization number is KK/2542-1/2023 (University of Pécs).

Total RNA was isolated from enterovirus-positive CSF samples and the accompanying faecal specimens using TRIzol LS and TRIzol Reagent (Thermo Fisher Scientific, Waltham, Massachusetts, USA), respectively, according to the manufacturer’s instructions. The conditions and reagents used in the subsequent RT-PCR reactions were the same as described previously [34], with minor modifications indicated below. For enterovirus-specific RT-PCR, complementary DNA (cDNA) was synthesized using MAXIMA H-minus Reverse Transcriptase (Thermo Fisher Scientific, MA, USA) and either UnivEnt-VP2-Rnew or UnivEnt-5UTR-Rnew reverse primers (Supplementary Table S1) [35], which were designed previously to recognize the VP2 or the 5’UTR genomic region, respectively, of the majority of the known enteroviruses. After cDNA synthesis, two separate PCR reactions were conducted using DreamTaq DNA Polymerase (Thermo Fisher Scientific, MA, USA), the same reverse primers used for cDNA synthesis, and a generic enterovirus forward primer (UnivEnt-5UTR-F, Supplementary Table S1) with a final primer concentration of 0.8 µM in a final volume of 25 µL. Temperature cycling of the PCR reactions was performed as follows: one cycle of 95 °C for 1 min, 39 cycles of denaturation at 95 °C for 20 s, annealing at a temperature 5 °C lower than the melting temperature of the primers for 20 s, and 72 °C for 1 min, followed by an additional extension step for 5 min at 72 °C.

Partial VP1 sequences were determined using various generic or E18-specific VP1 primer pairs in conventional or nested RT-PCR reactions (Supplementary Table S1) with the same conditions and reagents described above. In nested PCR reactions, the second round of PCR included 1 µl of the first-round PCR product in a final volume of 25 µL, and the thermal program had only 24 cycles.

The VP1 primers were designed based on a nucleotide sequence alignment of the most closely related E18 sequences identified by BLASTn searches using the 5’UTR and VP2 sequences determined in the enterovirus diagnostic assays. The nearly complete viral genome sequences of two selected E18 strains were determined by the primer-walking method, using sequence-specific and generic primers. PCR products were sequenced directly in both directions using a BigDye Termination Kit v1.1 (Thermo Fisher Scientific, MA, USA) and an automated sequencer (ABI 3500 Genetic Analyzer, Applied Biosystems, Hitachi, Japan).

GeneDoc ver. 2.7 software was used for sequence assembly [36]. Multiple sequence alignments were generated using the Multiple Sequence Comparison by Log-Expectation (MUSCLE) web tool (https://www.ebi.ac.uk/Tools/msa/muscle/). A maximum-likelihood phylogenetic tree with 1000 ultrafast bootstrap replicates was constructed using the IQ tree web server [37, 38], based on a nucleotide sequence alignment of the partial VP1 regions of all available E18 strains found in the NCBI database (accessed in October 2024), using the best-fit model (TVMe + G4), which was chosen using IQ-TREE multicore version 2.2.6 [39]. The tree was visualized using Interactive Tree Of Life (iTOL) v5 [40] and Corel Draw 2021.

A total of 82 (19.6%) CSF specimens were tested for CNS pathogens by BioFire FilmArray syndrome-specific multiplex PCR-based methods throughout the sampling year, and five (6.1%) of these (four from males and one from a female) tested positive for enteroviruses within a short, 3.5-month-long period between June 30 and October 12, 2023, confirming the (entero)viral acute CNS mono-infections. Using conventional RT-PCR and Sanger sequencing methods, four of the five enterovirus isolates (4277/2023/HUN, 4276/2023/HUN, 4675/2023/HUN, and 6833/2023/HUN; GenBank nos. PP861087-PP861090) were identified as E18 (Table 1) based on 580-nucleotide (nt)-long partial VP1, 537-nt-long partial 5’UTR, and 1,120-nt-long partial 5’UTR-VP2 sequences, and in the case of isolates 4276/2023/HUN and 6833/2023/HUN, the nearly complete genome sequences as well. Three (4277/2023/HUN, 4276/2023/HUN, and 4675/2023/HUN) of the four Hungarian strains (in cases 1–3, samples collected within 1 month between July 6 and August 6, 2023) had nearly identical (> 99%) partial VP1 gene sequences, while isolate 6833/2023/HUN exhibited up to 12% nucleotide differences when compared to the other three isolates. Using a BLASTn/BLASTp search of the GenBank database and phylogenetic analysis, the isolates 4277/2023/HUN, 4276/2023/HUN, and 4675/2023/HUN showed the highest nt (97.2–97.4%) and aa (98.81% and 98.95%) sequence identity and the closest phylogenetic relationship to the isolates EV18_Fr22_MAR8024 (OR840841) and EV18_Fr23_MAR5027 (OR840842), which were identified in pharynx and CSF specimens in France (GenBank, unpublished data) in October 2022 and July 2023, respectively (Fig. 1, Supplementary Fig. S1). However, 6833/2023/HUN had the highest nt (97%) and aa (98%) sequence identity and the closest phylogenetic relationship in VP1 to the E18 isolates 02/GDSZ/CHN/2019/R (MN688218) [41] and CHN/WX/CSF20 (OR095794) (GenBank, unpublished data) identified in China in April 2019 and October 2020, respectively (Fig. 1, Supplementary Fig. S1). In case 2, a faecal specimen was also available from the patient (Table 1), and it also tested positive for E18 by RT-PCR and sequencing (4275/2023/HUN, GenBank no. OR372160). There were four nucleotide differences (0.7%; one synonymous and three non-synonymous mutations) between the E18 sequences of the VP1 region identified in CSF (4276/2023/HUN) and faecal samples (4275/2023/HUN). All three non-synonymous mutations were located in the predicted EF-loop region of VP1 (data not shown). The nearly complete genome sequences (with a complete coding region and complete 3’UTR) of two selected E18 strains, 4276/2023/HUN and 6833/2023/HUN, showed 97.2% and 92.4% identity to the closest E18 strains EV18_Fr22_MAR8024 [OR840841] and CHN/WX/CSF20 [OR095794], respectively, identified by a BLASTn search. Interestingly, a significant drop in sequence difference was seen in the 2C/3A region of 6833/2023/HUN (Supplementary Fig. S2), which could indicate a previous recombination event in a recombination hotspot (2C/3A junction) or diverse mutation rates in different genome parts of 6833/2023/HUN. However, the putative recombination event was not supported by any of the recombination detection programs used (RDP, SimPlot), most likely due to the absence of a P3 donor sequence.

**Table 1:** Demographic and clinical characteristics of the patients with confirmed E18 infections

Case no.	Sex/age (year)	Location	Date of onset of symptoms	Potential source of the infection	Symptoms	Sample type	Date of sample collection and positive molecular test result for E18	E18 strain name and GenBank accession no.
Case 1	M/34	Villány	June 30, 2023	(Parent) contact with infected children, spouse with similar symptoms	Fever, headache, joint pain	CSF	July 6, 2023	4277/2023/HUN PP861088
Case 2	F/34	Pécs	July 13, 2023	Not known	Headache, retrobulbar pain, dizziness, nausea, photophobia	CSF	July 14, 2023	4276/2023/HUN PP861087
						Faeces	July 17, 2023	4275/2023/HUN OR372160
Case 3	M/34	Pécs	August 2, 2023	(Parent) contact with infected children; July 23–28: travel to Italy	Fever (max. 39°C), headache, lack of appetite, chills, retrobulbar pain, exanthema all around the body	CSF	August 6, 2023	4675/2023/HUN PP861089
Case 4	M/34	Pécs	October 12, 2023	Family member with fever	Fever (max. 40°C), headache, nausea, vomiting, limb pain	CSF	October 17, 2023	6833/2023/HUN PP861090

M, male; F, female; CSF, cerebrospinal fluid; E18, echovirus type 18

**Fig. 1:**
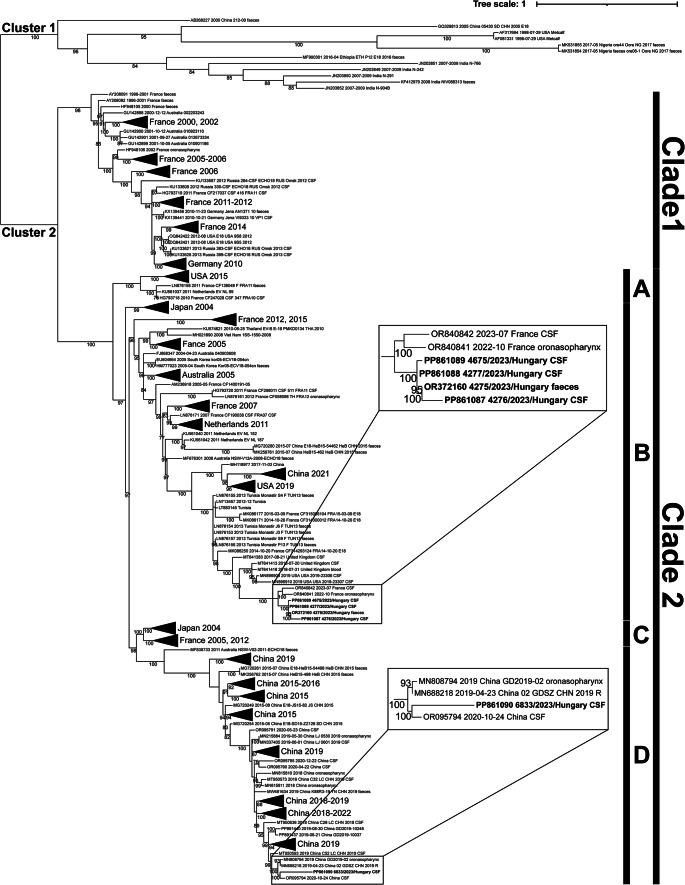
Phylogenetic analysis based on 580-nt-long partial nucleotide sequences of the VP1 region of the E18 viruses (N = 334) with sequences available in the GenBank database. The strains from this study are indicated by bold letters. E18 strains form two phylogenetic clusters (cluster 1 and cluster 2). Cluster 2 is divided into clades (indicated with black and grey lines) that correlate well with the geographical distribution and temporal appearance of certain E18 strains. Genetically highly similar strains that originated from the same geographic area are collapsed into a black triangle. The complete phylogenetic tree can be found in Supplementary Figure S1

According to the patients’ medical history, all four of the hospitalized adult patients were born in 1989 (Table 1) and had no known immunodeficiency or underlying diseases. Three of them were residents of the city of Pécs, which is in the centre of Baranya County, and the fourth lived in a small town (Villány) ~ 30 km from Pécs. The most common symptoms were headache, fever, retrobulbar pain, nausea, and joint and limb pain. Exanthema, photophobia, and vomiting were less frequent (Table 1). Approximately one week prior to the onset of symptoms, three of the patients had had contact with children living in the same household (children/parents relationship, Table 1) who had had fever, diarrhoea, or maculopapular rash, and in one case, the spouse in the same household had had symptoms similar to those of the hospitalized patient (Table 1). These affected children and the spouse did not require hospitalization or treatment, and no microbiological tests were performed. No direct epidemiological link could be detected between the confirmed E18-infected adults. The four adult patients had an unremarkable neurologic status, as assessed by physical examination. CT, EEG, and MR imaging did not reveal any pathological abnormalities. All of the patients had a slightly elevated (16.7–25.8 mg/ml) C-reactive protein (CRP) level (Table 2). Only one patient (case 2) had leucocytosis, which was detected on the first day after the onset of symptoms and resolved a day later. Two of the patients (cases 3 and 4) had lymphocytopenia, but two of them (cases 1 and 2) had a normal lymphocyte count. CSF laboratory parameter values corresponded to serous meningitis: an elevated CSF protein level in two cases and elevated white blood cell and mononuclear cell counts in four cases (Fig. 2). The highest total CSF protein concentration was 846 mg/l (case 2). However, the protein levels in case 3 and case 4 were in the normal range (Fig. 2). The average CSF white blood cell count was 263 cells/mm^3^, which is 38.2–67 times higher than the normal limit. The average CSF mononuclear cell count was 150 cells/mm^3^, with the highest value recorded in case 2 (313 cells/mm^3^, 62.6 times higher than the normal limit) and the lowest value recorded in case 4 (23 cells/mm^3^, 4.6 times higher than the normal limit). The average CSF polymorphonuclear cell count was 113 cells/mm^3^, with the highest value recorded in case 3 (177 cells/mm^3^, 88.5 times higher than the normal limit) and the lowest value recorded in case 2 (22 cells/mm^3^, 11 times higher than the normal limit) (Fig. 2). All of the patients received supportive treatment and were dismissed from the hospital in good condition without residual symptoms. The length of hospitalization was 1–6 days.

**Table 2:** Serum laboratory parameters of the confirmed E18 cases. Values outside the normal range are in bold

No	Time of laboratory testing	CRP normal range: < 5 mg/ml	WBC normal range: 4.0–10.0 G/L	Neutrophil cell count normal range: 1.78–5.38 G/L	Lymphocyte cell count normal range: 1.3–3.57 G/L
Case 1	6th day after onset of symptoms	**24.5** mg/ml	4.59 G/L	1.81 G/L	1.94 G/L
Case 2	1st day after onset of symptoms	**23.3** mg/ml	**11.35** G/L	**7.94** G/L	2.49 G/L
Case 3	4th day after onset of symptoms	**16.7** mg/ml	5.76 G/L	4.28 G/L	**1.03** G/L
Case 4	2nd day after onset of symptoms	**25.8** mg/ml	6.37 G/L	4.94 G/L	**0.79** G/L

CRP, C-reactive protein; WBC, white blood cells; G/L = 10^9^/liter

**Fig. 2:**
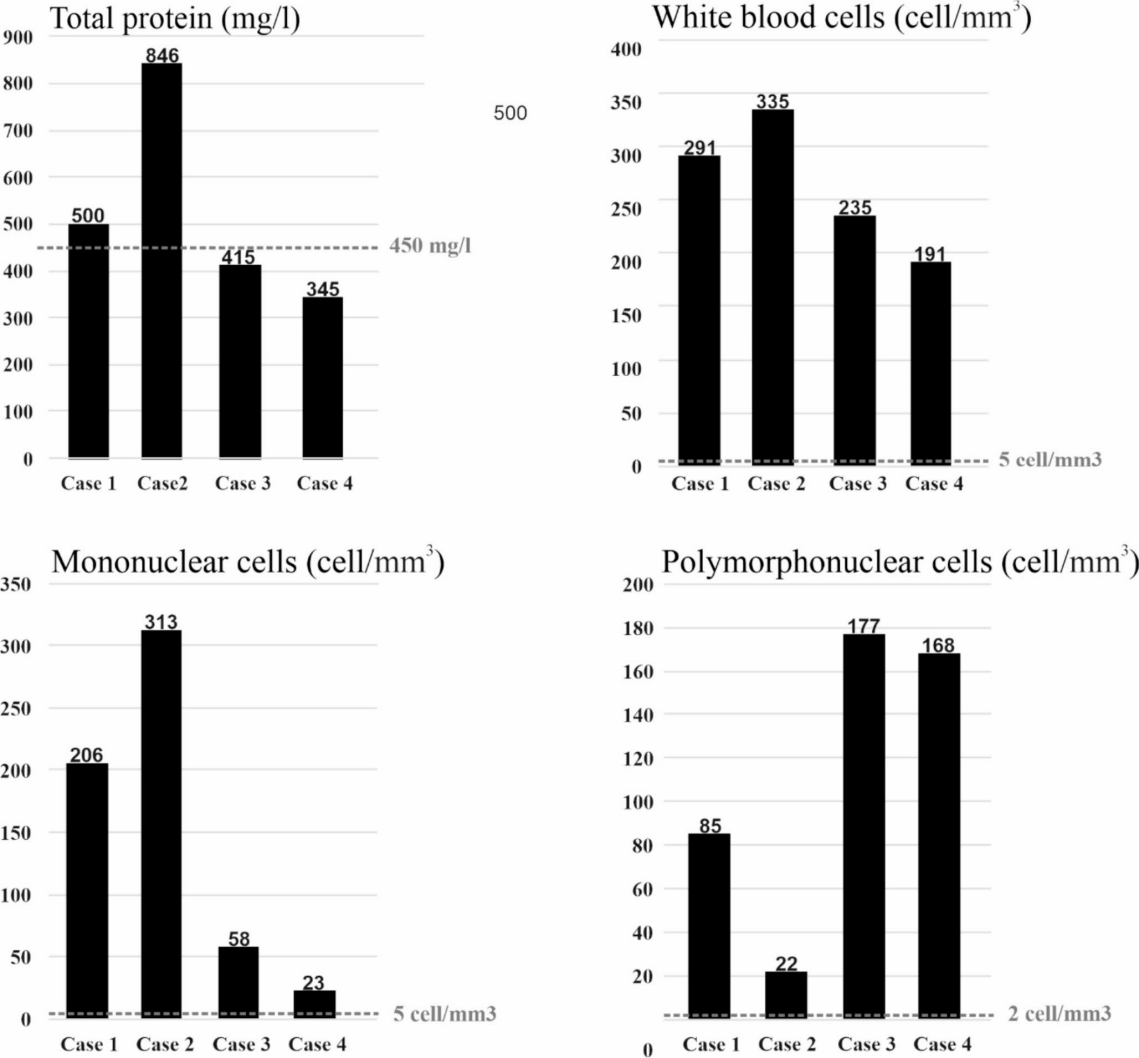
Laboratory parameters (total protein, white blood cells, mononuclear cells, and polymorphonuclear cells) of cerebrospinal fluid (CSF) specimens collected from adults (N = 4) with encephalitis with E18 infections. The dashed line indicates the upper limit of the laboratory normal range in each section

Here, we report four cases of confirmed echovirus type 18 (E18) CNS infection with meningoencephalitis, identified by syndrome-specific molecular microbiological laboratory methods, sequence analysis and phylogenetic analysis. All of the cases occurred within a narrow geographic region and within a 3.5-month period in mid-2023. Although E18 is believed to primarily infect neonates and young children [4, 15], in our study, adult patients of the same age became ill and were hospitalized. Since the majority of enterovirus infections are asymptomatic or have a mild course, and, based on our knowledge so far, are rarely accompanied by CNS complications [4], we assume that a larger number of E18 infections may have occurred in our region that were not clinically diagnosed. This is also supported by the fact that three of the infected adults had been in contact with children or family members showing typical symptoms of enterovirus infection prior to their illness, and these could have been the sources of the infections. In addition, three of the four E18 isolates were found to have very similar nucleotide sequences. Based on these results, we hypothesize that an unusual local/regional outbreak of E18 (or a certain lineage of E18) occurred in south-western Hungary in 2023. Cases of encephalitis with a similar course were observed in a neighbouring county (Somogy) during the same time period, but no laboratory diagnosis was obtained in those cases (Kiss G., head of the epidemiology department, personal communication).

The most common disease conditions associated with E18 are meningitis, encephalitis, diarrhoea, and exanthema. E18-associated hand, foot, and mouth disease, an unusual clinical manifestation, has been reported in Asia in recent years [17, 24, 25, 27, 28, 42]. In our focused group of patients with potential CNS infections, adult patients had headache, fever, retrobulbar pain, nausea, joint and limb pain, vomiting, photophobia, and exanthema. The serum laboratory parameters of the patients showed slightly elevated CRP levels, an elevated leucocyte concentration in one case (case 2), and lymphocytopenia in two cases (cases 3 and 4). However, cerebrospinal fluid laboratory parameters were characteristic of viral meningitis except in cases 3 and 4, where polymorphonuclear cells were dominant in CSF samples and mononuclear cell counts were elevated, which is an atypical finding [43].

Echovirus 18 is spreading globally. However, since 2013, there has been a decrease in sample collection data from E18-associated outbreaks in Europe compared to the previous period [11–13, 16–18, 20], and we found no literature data on the genotyping of E18. Based on our phylogenetic analysis of the VP1 region, the E18 VP1 capsid nucleotide sequences in the GenBank database can be grouped into at least two main phylogenetic clusters worldwide: clusters 1 and 2 (Fig. 1; Supplementary Fig. S1). Cluster 2 can be divided further into two clades (clades 1 and 2). Members of clade 1 circulated from 1996 to 2014 in France, Germany, Russia, Australia, and the USA. Clade 2 is further divided into four subclades (A-D) and includes strains reported between 2004 and 2023. Clade 2A consists of strains that circulated in France, the Netherlands, and the USA between 2010 and 2015. Clade 2B strains circulated in Europe, Asia, North Africa, and Australia between 2004 and 2023. Clade 2C consists of strains from Japan and France between 2004 and 2012. Finally, clade 2D strains were predominantly identified in China between 2015 and 2020 (Fig. 1; Supplementary Fig. S1). The Hungarian E18 strains reported here are clustered within different subclades of clade 2 (C and D) and are closely related to French strains (unpublished data) reported in 2022 and 2023, as well as a Chinese strain reported between 2019 and 2020 [41] (GenBank, unpublished data). This means that members of two different subclades of E18 clade 2 could be detected simultaneously in our region that potentially originated from different geographic regions. Currently, a retrospective molecular epidemiological study of the circulation of E18 variants in Europe in 2023 is ongoing within the framework of the European Non-Polio Enterovirus Network (ENPEN, https://escv.eu/european-non-polio-enterovirus-network-enpen/). Preliminary data indicate that the total number of confirmed E18 infections in Europe increased significantly in 2023 (Harvala H., personal communication, unpublished data). Based on our preliminary phylogenetic analysis, we suggest criteria for subgenotyping of different E18 variants that take different genomic regions into consideration to monitor the spread of different subgenotypes/recombinants associated with local outbreaks.

The following limitations of this study should be taken into account: 1) The sensitivity of the syndrome-specific multiplex-PCR-based method for enteroviruses including genetically diverse E18 strains is unknown, and false-negative test results are possible. 2) Only some of the hospitalized patients were tested, and there are no data on the incidence of E18 infections in the general study population in mid-2023. 3) Identification and publication of additional European E18 strains would be necessary to confirm the E18 epidemic at the European level.

Based on the results of this study, continuous and systematic – retrospective and prospective – investigations are necessary to explore the circulation and outbreaks of variants of the E18 picornavirus in different risk and clinical groups of the human population in Europe and to identify potential epidemic strains over time.

## Electronic Supplementary Material

Below is the link to the electronic supplementary material

Supplementary Material 1

Supplementary Material 2

## Data Availability

The nucleotide sequence data reported here are available in the DDBJ/EMBL/GenBank databases under the accession numbers OR372160 and PP861087-PP861090.
