# Supplementary figures and images for: A meningoencephalitis outbreak associated with echovirus type 18 (E18) in south-western Hungary in mid-2023
Source: Arch Virol. 2024 Nov 4;169(11):237. doi: 10.1007/s00705-024-06166-5 (PMC11534849; doi:10.1007/s00705-024-06166-5)

**Figure S2.**


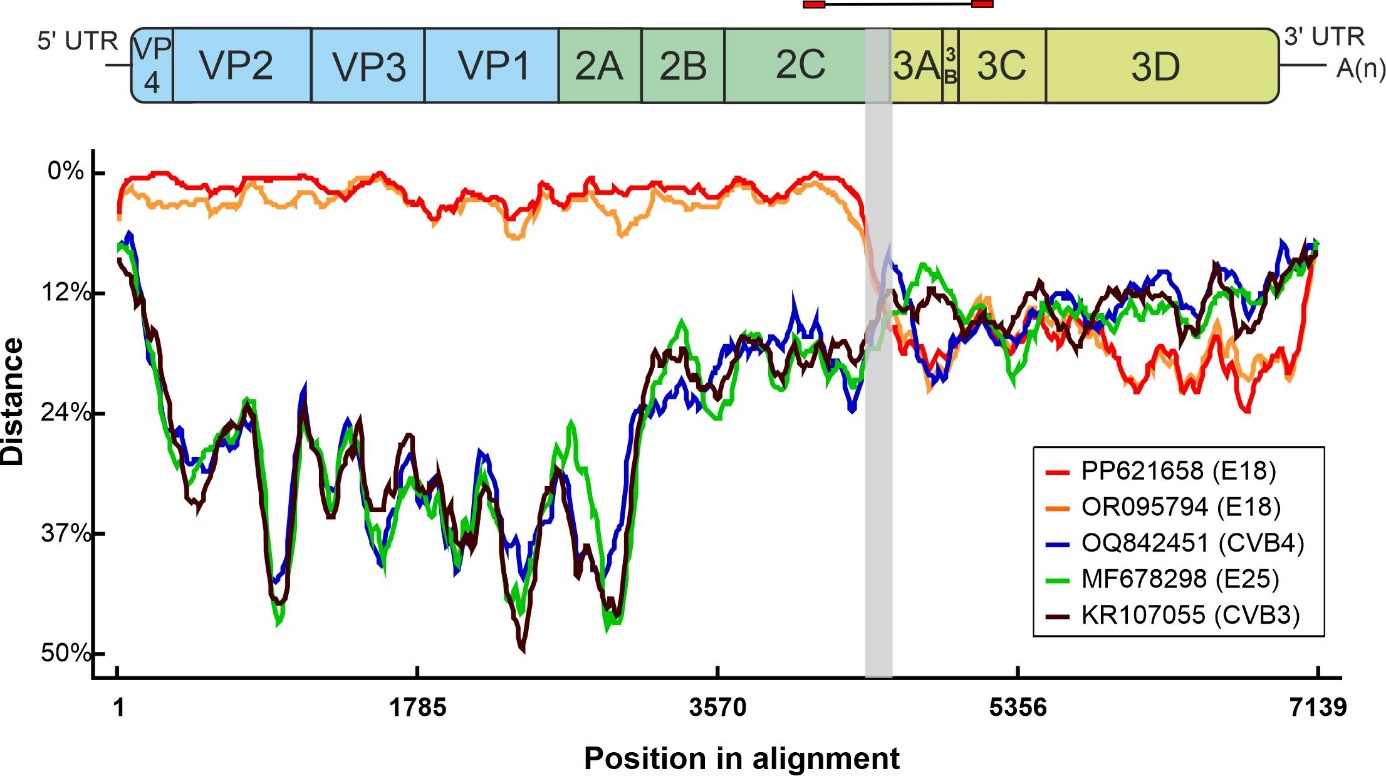

Supplement: Supplementary file 1 — Supplementary Material 1 [file 705_2024_6166_MOESM1_ESM.docx]
