# Supplementary material for: A meningoencephalitis outbreak associated with echovirus type 18 (E18) in south-western Hungary in mid-2023
Source: Arch Virol. 2024 Nov 4;169(11):237. doi: 10.1007/s00705-024-06166-5 (PMC11534849; doi:10.1007/s00705-024-06166-5)
Supplement: Supplementary file 2 — Supplementary Material 2 [file 705_2024_6166_MOESM2_ESM.docx]

**Table S1**: List and features of oligonucleotide primers designed and used in this study. The sequences of primers designed for whole genome determination are provided by the authors upon request. Abbreviations: RT-nPCR: RT- nested-PCR; E18: echovirus type 18; VP: viral protein; UTR: untranslated region

| **Target virus** | **Target genomic region** | **Reaction type** | **Primer name** | **Primer sequence (5' - 3')** | **Primer reference** | **Product length (bp)** |
| --- | --- | --- | --- | --- | --- | --- |
| Enterovirus sp. | 5'UTR/VP2 | Enterovirus diagnostic RT-PCR | UnivEnt-5UTR-Rnew* | ATT GTC ACC ATW AGC AGY CA | this study | 537*/1,120# |
|  |  |  | UnivEnt-VP2-Rnew# | GGD AAY TTC CAC CAC CAN CC | this study |  |
|  |  |  | UnivEnt-5UTR-F | GTA CCY TTG TRC GCC TGT T | [43] |  |
| echovirus type 18 (E18) | VP1-2A | E18 typing RT-PCR | E18-VP1-Rgen | TCC CAC ACR CAR TTY TGC CAG TC | this study | 1,156 |
|  |  |  | EV18-VP1-Fgen | GTI TTG TGC GTT CCY TGG ATT AG |  |  |
|  | VP1 | E18 typing RT-nPCR  (1st round) | E18-VP1-Rout | GGG TGY GGT GTG TCA GTG ATG | this study | 870 |
|  |  |  | E18-VP1-Fout | CAC CCT TCA TGG CAC AAC CTG | this study |  |
|  |  | E18 typing RT-nPCR (2nd round) | E18-VP1-Fin | CCA GCG ACA CCC TAC AAA C | this study | 580 |
|  |  |  | E18-VP1-Rin | ATA GTG CTA GTG ATT TGG TGG G | this study |  |
